# Stiffening Performance of Cold-Formed C-Section Beam Filled with Lightweight-Recycled Concrete Mixture

**DOI:** 10.3390/ma15092982

**Published:** 2022-04-20

**Authors:** Ahmed W. Al Zand, Mustafa Farooq Alghaaeb, Mohammed Chyad Liejy, Azrul A. Mutalib, Riyadh Al-Ameri

**Affiliations:** 1 Department of Civil Engineering, Universiti Kebangsaan Malaysia (UKM), Bangi 43600, Malaysia; eng.m0123456@gmail.com (M.F.A.); chyd1982@gmail.com (M.C.L.); azrulaam@ukm.edu.my (A.A.M.); 2 Engineering Affairs, Ministry of Culture, Baghdad 13032, Iraq; 3 Al-Hawija Technical Institute, Northern Technical University, Kirkuk 36001, Iraq; 4 School of Engineering, Faculty of Science Engineering and Built Environment, Deakin University, Geelong, VIC 3216, Australia; r.alameri@deakin.edu.au

**Keywords:** stiffening C-section, lightweight-recycled concrete, flexural strength, cold-formed beam, concrete filled beam, confinement

## Abstract

The aim of this paper is to investigate the flexural performance of a new steel–concrete composite beam system, which is required to carry higher loads when applied in flooring systems with less self-weight and cost compared with conventional composite beams. This new composite member is prepared by filling a single cold-formed steel C-section with concrete material that has varied lightweight-recycled aggregates. In addition, varied stiffening scenarios are suggested to improve the composite behavior of this member, since these cold-formed C-sections are of a slender cross-section and more likely to buckle and twist under high bending loads than those of hot-rolled C-sections. The influence of using four different lightweight-recycled aggregates that combine together in the infill concrete material was investigated. These recycled aggregates are recycled concrete aggregate (RCA), expanded polystyrene (EPS) beads, crumb rubber aggregates (CRA) and fine glass aggregates (FGA). For this purpose, 14 samples of cold-formed galvanized steel C-purlin were filled with concrete material (containing 0 to 100% recycled aggregates) which are experimentally tested under pure bending load, and 1 additional sample was tested without the filling material. Further numerical models were prepared and analyzed using finite element analysis software to investigate the effects of additional parameters that were not experimentally examined. Generally, the results confirm that filling the C-sections with concrete material that contains varied percentages of recycled aggregates offer significantly improved the flexural stiffness, bending capacity, and ductility performances. For example, using infill concrete materials with 0% and 100% recycled aggregate replacement increased the bending capacity of hollow C-section by about 11.4 and 8.6 times, respectively. Furthermore, stiffening of the concrete-filled C-sections with steel strips or screw connectors eventually improved the composite behavior of the specimens which led to an increase in their bending capacities accordingly, and this improvement enhanced more with an increased number of these strips and connectors.

## 1. Introduction

In recent years, several comprehensive studies have investigated the performance of composite concrete-filled hollow steel tube members, and have reported significantly improved strength, stiffness, and ductility [1]. For example, the bending capacity of a hollow steel tube beam has been enhanced by using concrete as the infill material, such as circular concrete-filled steel tube (CFST) beam and column [2], elliptical CFST beam and column [3], rectangular CFST members [4], CFST and RC columns under combination of compression and bending loads [5], square and rectangular steel tube filled with normal and high strength concrete [6], built-up tubes filled with concrete [7,8,9], and concrete-filled steel profiled wall and slab systems [10,11]. Generally, the cost of one ton of cold-formed steel C-sections is about 800–900 USD as compared with 1100–1200 USD per ton for the equivalent hot-rolled C-sections, whereas normal concrete material is about 40–50 USD per ton. For example, in Malaysia’s local market, engineers have adopted cold-formed steel C-sections in systems of concrete-filled steel tube members as a comparable solution to hot-rolled C-sections. Cold-formed steel C-sections filled with concrete can increase the member’s self-weight from about seven to eight times, as well as strength capacity by more than four times [12].

The slender cross-section of steel tube members filled with concrete are usually lighter and cost less than those with non-compact and compact sections, and they are more favorable in construction projects. However, these slender tube sections are more likely to buckle under the compression stress that occurs due to bending load, such as with cold-formed steel tubes filled with normal and recycled concrete [12], or when testing the slenderness limit of cold-formed circular hollow tube [13], predicting the flexural stiffness and strength of CFST beams [14], studying the post-cracking behavior of hybrid fiber-reinforced CFST beams [15], or investigating the slenderness limits of circular and rectangular CFST beam [16,17]. Therefore, several studies have used different scenarios to investigate the stiffening performance of slender tube sections in order to delay/restrict outward buckling failure [18,19,20,21,22]. For example, comprehensive experimental and numerical investigations have been conducted by Al Zand et al. (2020) for stiffening a highly slender square steel tube beam filled with normal concrete by using internal steel stiffeners welded along the tube’s sides (web and flanges) [19] or by providing a V-grooved shape to improve the plate’s resistance against buckling behavior [20]. The flexural performance, including bending and energy absorption capacities, of the stiffened composite members was significantly improved by more than 20%, and was further improved by increasing the number of stiffeners, because the local buckling of tubes was sufficiently delayed due to the effects of the internal/external stiffeners. However, using additional steel processes such as welding [19] or fabricating new cross-sections [20], and/or adding additional steel parts for stiffening the concrete-filled steel tube beams [23] can increase costs and time which have impacts on projects. Therefore, steel C-sections (C-purlins) fabricated with a thinner thickness and lower self-weight than equivalent hot-rolled steel C-sections have recently been adopted in systems of concrete-filled steel tube members [12,24]. In particular, combining two C-purlins face-to-face to produce a slender hollow steel tube beam filled with concrete material and internal steel stiffeners (top and bottom lips of the C-purlin) has achieved significant flexural performance under pure bending loads [12,24]. A single C-purlin in a concrete-filled tube composite system shows different failure modes than two C-purlins joined together, because it is a challenge to confine the infill concrete specifically along the open side of the C-purlin, where the core concrete could leak out the section.

Furthermore, engineers have given significant attention to reducing the cost and self-weight of concrete-filled steel tube members by adding different types of waste materials such as recycled aggregates in concrete mixtures [9,18,25,26,27,28,29,30]. Several researchers have experimentally investigated the performance of steel tube members by using recycled concrete aggregate, usually known as “RCA or RAC”, prepared from crushed concrete elements of demolished old reinforced concrete structures; the raw coarse aggregate of the infill concrete material inside steel tube members was partially replaced with RCA (by volume) [5,12,26,27,30,31,32,33,34]. In general, the performance testing results of steel tube beams filled with recycled concrete material are quite close to those of corresponding members filled with normal concrete material, with slightly less strength capacity [26,31]. Moreover, lightweight concrete material has been adopted as infill in a system of concrete-filled steel tube beams/columns in order to reduce the self-weight of the core concrete part of these composite members [35,36,37,38]. One of the most useful lightweight aggregates used to reduce the self-weight of concrete mixtures has been expanded polystyrene (EPS) beads, which have been used to replace the raw coarse aggregate of the concrete mixture by volume [12,19,39,40,41]. Additionally, waste materials such as recycled aggregates have also been used in concrete mixtures of plain and reinforced concrete elements such as crumb rubber aggregates (CRA) [42,43,44] and fine glass aggregates (FGA) [45,46,47,48,49]; however, they have not yet been used in composite members. Therefore, it is suggested that using more than two types of waste materials (RCA, EPS, CRA, and/or FGA) as recycled aggregates in a system of concrete-filled steel tube members is expected to be useful and could be considered to be a new concept for environmental improvement.

Based on the above literature, the main aim of this research is to investigate the flexural performance of single C-sections filled with concrete materials. This new composite beam system is suggested to achieve a lighter weight and lower cost than the conventional composite beams system, which is required to be adopted into flooring systems since, to date, cold-formed C-sections are applicable for a roofing system to carry lighter loads compared to those applied on floors. Therefore, 15 specimens were prepared and tested under four-point bending. The specimens were categorized into four groups: Group 1, to study the flexural performances of a cold-formed C-section (C-purlin) filled with concrete material containing different percentages of combined recycled aggregates which are RCA, EPS, FGA, and CRA; Group 2, to investigate the influence of using multi-steel strips for restraining the top and bottom flanges together along the open side of the C-sections to improve the concrete’s confinement behavior; and Groups 3 and 4, to investigate the effects of providing connectors on the top-half of the C-section’s cross-section along varied span lengths. Furthermore, finite element (FE) models were built and analyzed to further investigate the flexural performance of the newly proposed steel C-sections filled with concrete material. The results obtained from the current experimental and numerical works are discussed.

## 2. Experimental Approach

### 2.1. Specimens Preparation

**Figure 1:**
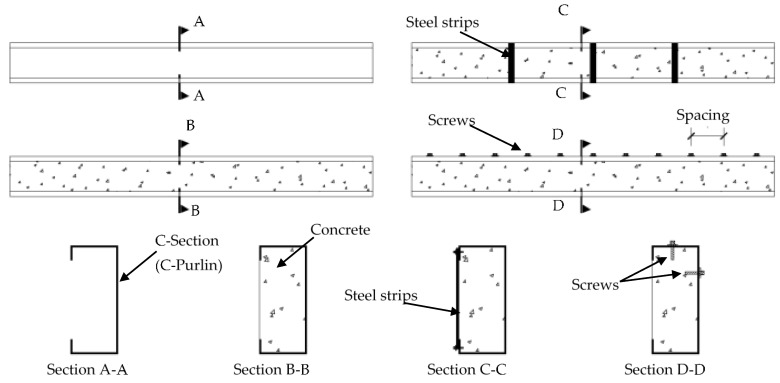
Sketch of suggested specimens with C-sections filled with concrete (out of scale).

**Figure 2:**
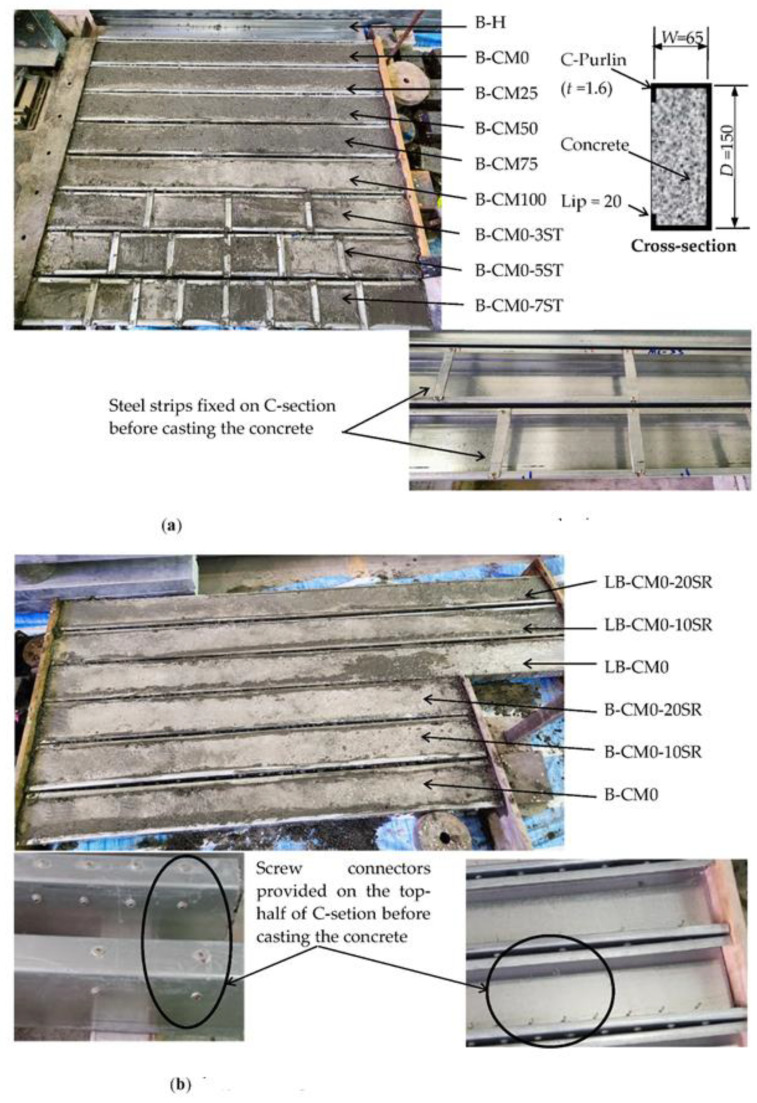
Specimens of C-sections filled with concrete: (**a**) concrete filled C-section specimens with/without steel strips; (**b**) short and long specimens stiffened with screw connections.

Fifteen (15) specimens were prepared in this research by filling single C-sections (galvanized steel C-purlins) with concrete material to investigate their flexural performance. In addition, to improve the behavior of the composite C-sections and core concrete, the top and bottom flanges were stiffened with steel strips by providing multi-screw connectors along the top-half of a C-section’s cross-section, as shown in Figure 1. The C-sections had all been fabricated with depths of 150 mm, flange widths of 65 mm, lips of 20 mm, and thicknesses of 1.6 mm. Twelve of the specimens were prepared with lengths of 1.5 m and, three specimens were prepared with 2.0 m lengths. Group 1 consisted of 6 specimens designed to study the effects of concrete filling material that contained varied waste materials (RCA, EPS, CRA, and FGA); one specimen in this group was tested as a control hollow beam (B-H, see Section A-A in Figure 1), and the other 5 specimens were tested with different percentages of recycled aggregate in the concrete mixtures equal to 0%, 25%, 50%, 75%, and 100% (identified as specimens B-CM0, B-CM25, B-CM50, B-CM75, and B-CM100, respectively, see Section B-B in Figure 1). Group 2 consisted of 3 specimens designed to study the effects of externally stiffening the open side of the concrete-filled C-section beam with 3, 5, and 7 steel strips (identified as B-CM0-3ST, B-CM0-5ST, and B-CM0-7ST, respectively, see Section C-C in Figure 1); all specimens in this group were filled with normal concrete (0% recycled aggregates). Group 3 consisted of 3 specimens designed to study the effects of using multi-screw connectors that were provided before concrete casting and distributed along 10 and 20 equal spacing (identified as B-CM0-10SR and B-CM0-20SR, respectively, see Section D-D in Figure 1), as well as 1 specimen without screw connectors (B-CM0). Finally, Group 4 was the same as Group 3 (3 specimens) but with longer span lengths equal to 2.0 m (identified as LB-CM0, LB-CM0-10SR, and LB-CM0-20SR). All specimens were placed horizontally on their backsides during concrete casting, and both sides of the specimens were temporarily sealed to prevent concrete leaking, as shown for all specimens in Figure 2. The specimens were cured at room temperature until the day of testing (after 30 days from casting day). Table 1 presents the designations, size, and physical properties of the specimens.

### 2.2. Material Properties

**Figure 3:**
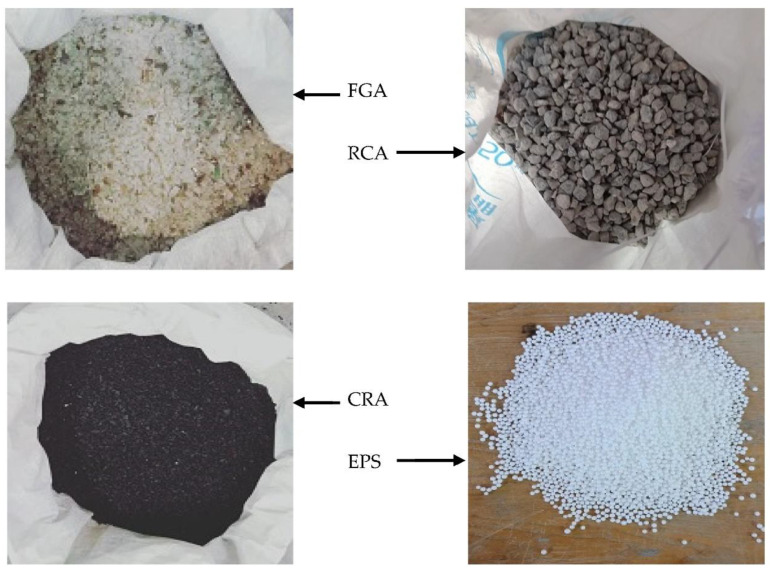
Recycled aggregate materials used in the concrete mixture.

The C-sections used in this research were supplied from the same batch and had been previously tested by Al Zand et al. [12], with average yield strength, ultimate strength, and elastic modulus values equal to 489 MPa, 558 MPa, and 201 GPa, respectively. The concrete mixtures contained different percentages of four recycled aggregates that replaced the raw aggregates by volume, as shown in Figure 3. The recycled aggregates were: (a) EPS beads with sizes ranging from 4 to 6.3 mm and density equal to 9.5 kg/m^3^ (replaced raw coarse aggregate), (b) RCA prepared from crushed old concrete elements with 5–19 mm particle sizes and overall density equal to 1278 kg/m^3^ (replaced raw coarse aggregate), (c) CRA prepared from crumb rubber with density equal to 600 kg/m^3^ and 2–5 mm particle sizes, and (d) FGA prepared from crushed waste glass material with density equal to 1570 kg/m^3^ and 1–5 mm particle sizes (both CRA and FGA replaced raw fine aggregate by volume). For each concrete mix designed in this research, 3 cubes (150 mm) were taken during the casting day which were cured and tested at 28 days, according to the standard of BS 1881:1983. Table 2 presents the proportion of concrete mixture (CM) including the percentages (0, 25%, 50%, 75%, and 100%) of combination from four different recycled aggregates (RCA, EPS, CRA, and FGA) that were used in each mixture. Also, the values of physical properties (density, cubic compressive strength, *f_cu_*, and elastic modulus, *E_c_*) of each concrete mixture are shown in the same table.

### 2.3. Test Setup

**Figure 4:**
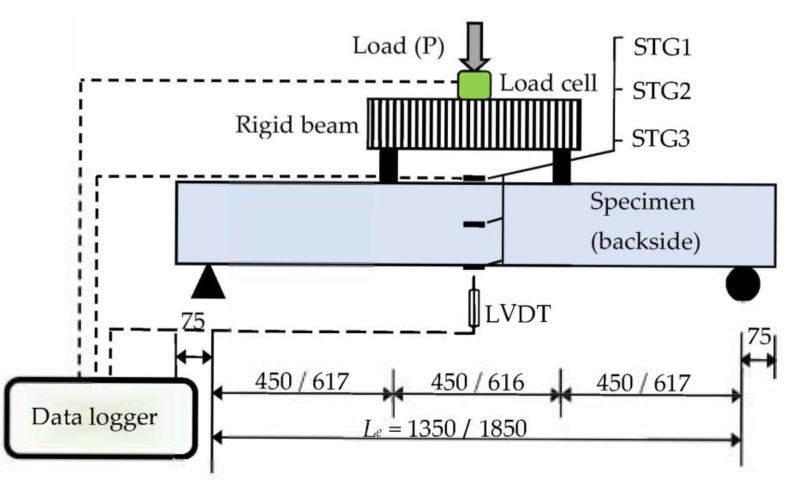
Schematic test setup for short/long specimens (all dimensions in mm).

The specimens were tested under pure bending load (two-point load), as shown in Figure 4. The static load was continually applied using a 500 kN capacity manual hydraulic jack with a loading rate of about 5–7 kN/minute. Single linear variable differential transducers (LVDT) were placed underneath the mid-span of specimens. Three strain gauges were fixed at the top flange (STG1), mid-depth (STG2), and bottom flange (STG3) of the steel C-section specimens to read the changes in tensile strain during the test. The data collected from the LVDT, load cell, and strain gauges during the testing were saved in a computer equipped with a data logger device (see Figure 4).

## 3. Test Results Discussion

### 3.1. Failure Modes

**Figure 5:**
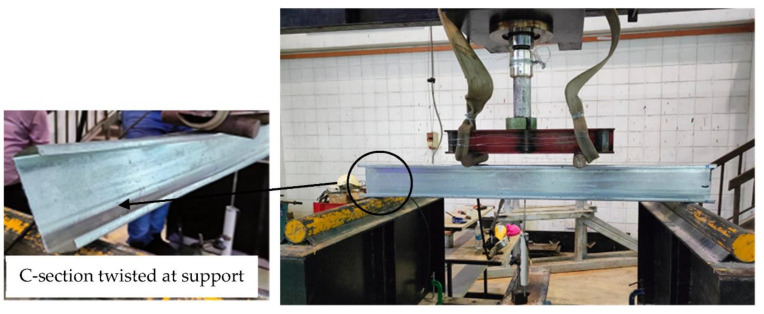
Typical failure modes of hollow C-section specimen.

**Figure 6:**
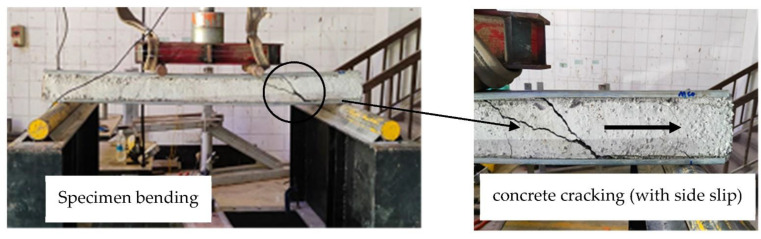
Typical failure modes of C-section specimen filled with normal concrete.

**Figure 7:**
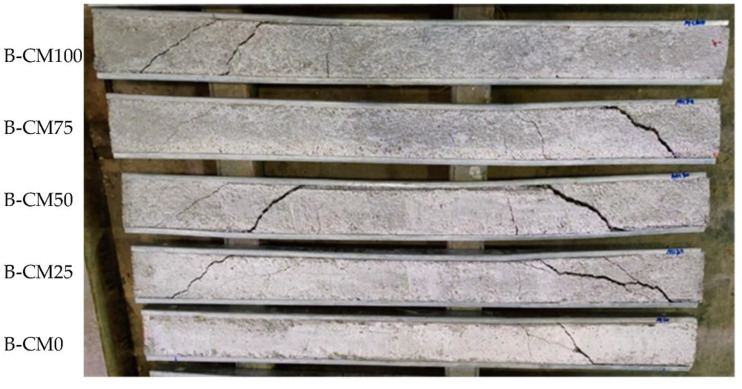
Typical failure modes of recycled concrete-filled C-section specimens.

In this section, we present and discuss the failure modes that were recorded from the experimental testing of the specimens of steel C-sections filled with concrete materials, where all specimens were tested beyond their ultimate capacities in order to further investigate extreme behavior. First, the hollow C-section showed twisting failure for the webs at the supports even before reaching its ultimate bending capacity, as shown in Figure 5. However, the same C-section, when filled with concrete materials (B-CM0), showed delaying in the twisting failure at the supports, where the web was twisted at higher loading stage compared to that with hollow section. The tested specimen B-CM0 showed normal deflection behavior (almost half-sine wave deflection behavior) very similarly to the conventional concrete-filled cold-formed steel tube beam [12], as shown in Figure 6. Generally, the deflection of the concrete-filled specimen (B-CM0) increased continually with increases in the applied load until concrete cracks started to occur in the high shear zone (the distance between the point load and adjacent support). Then, these cracks further increased, and the concrete part slipped outside the C-section at the extreme loading stage (see Figure 6). Replacing the infill concrete material in the C-sections with different percentages of recycled aggregates showed failure modes similar to that of the C-section filled with normal concrete (0% recycled aggregates), even for the specimen filled with 100% recycled aggregates concrete materials, as shown in Figure 7. Furthermore, the failure mode of the single hollow C-section showed different failure behavior than that of double hollow C-section which showed buckling of the entire cross-section at the supports [12]. Meanwhile, the concrete filled single C-section showed almost the same failure behavior of the double C-section filled with concrete materials [12], where both deflected smoothly and behaved similarly to the half-sine wave and their top flanges buckled at the extreme loading stage. The difference is that the single C-section showed less concrete confinement behavior, thus the concrete was widely cracked and slid out the steel tube at the extreme loading limit (see Figure 6), which did not occur when double C-sections were used [12].

**Figure 8:**
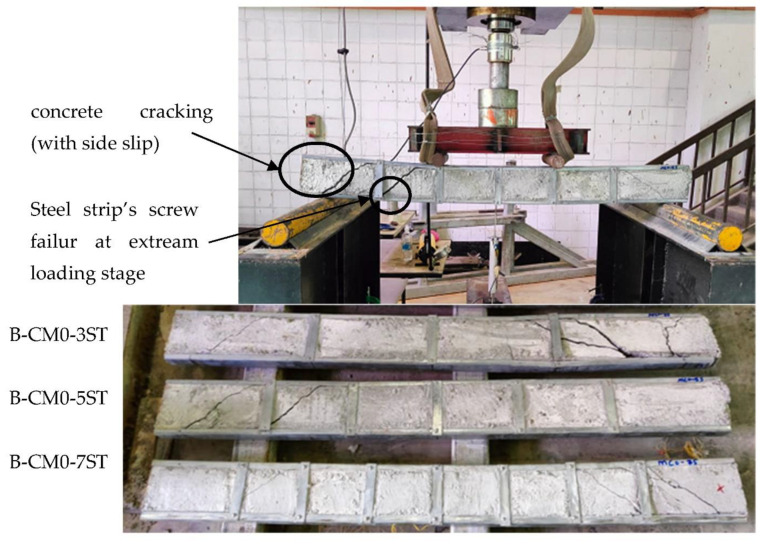
Typical failure modes of stiffened concrete filled specimens with steel strips.

In general, the concrete-filled C-section specimens stiffened with multi steel strips (i.e., B-CM0-3ST, B-CM0-5ST, and B-CM0-7ST) showed the same behavior as the unstiffened specimen (B-CM0), as shown in Figure 8. However, the appearance of concrete cracks in the stiffened specimens was delayed and occurred at a higher loading stage than that of the unstiffened specimen B-CM0, this is due to the influence of these steel strips that restricted the top and bottom flanges from opening under high bending which led to an improvement in the behavior of concrete confinement. Increasing the number of steel strips further enhanced the concrete confinement behavior. In this experimental study, the screw connectors located at the maximum shear zone (between the point load and adjacent support) failed under the extreme loading stage, and the concrete cracks suddenly increased accordingly (see Figure 8). Therefore, the performance of these steel strips can be more enhanced by providing better connection to the top and bottom flanges of the single cold-formed C-section, in order to better improve the confinement behavior of the core concrete of the newly suggested composite beams system.

**Figure 9:**
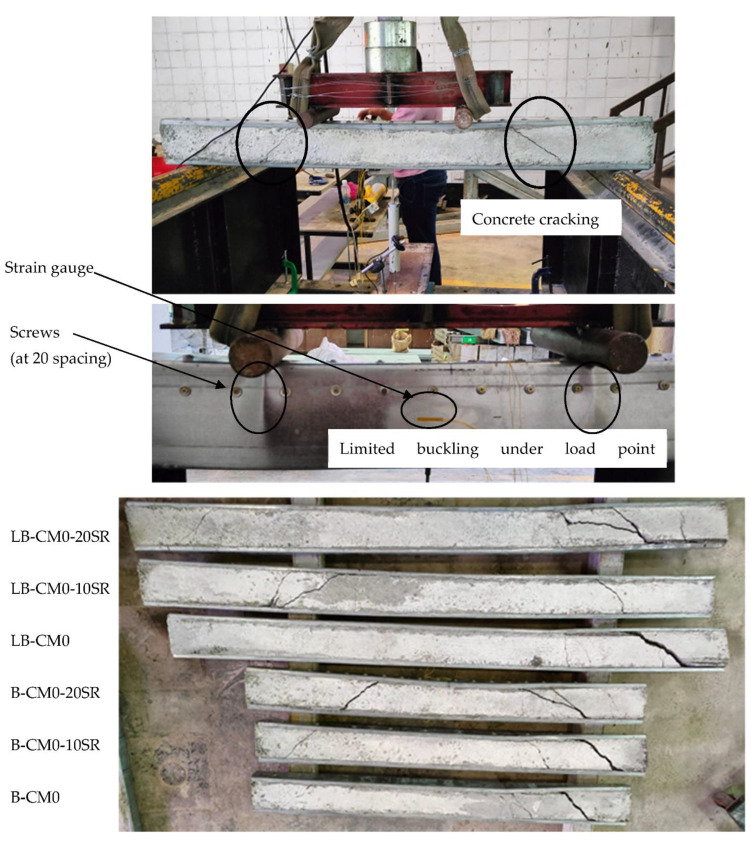
Typical failure modes of stiffened concrete filled specimens with screws.

Figure 9 shows the failure mode of the tested specimens that were stiffened with multi-screw connectors provided along the top-half of a C-section’s cross section. The concrete cracks began at the high shear zone similar to the unstiffened specimen (B-CM0). However, for the stiffened specimens with screw connectors, the side-slip failure of the cracked concrete part from the C-section was limited compared to control specimen B-CM0. This behavior occurred due to the influence of these connectors, which are well bonded with the core concrete and tilted perfectly to the body (top flange and web) of the steel C-section. Accordingly, increasing the number of these screw connectors led to more improvement in the interaction between this composite beam’s parts (core concrete and steel C-section), and also delayed/restricted the steel tube’s outward buckling behavior at the maximum bending zone (between both point loads and/or under the point loads). Lastly, increasing the span of concrete filled C-section specimen stiffened with multi-screw connectors had not much of a difference to the corresponding specimen with shorter span (see Figure 9).

### 3.2. Load vs. Deflection Relationship

**Figure 10:**
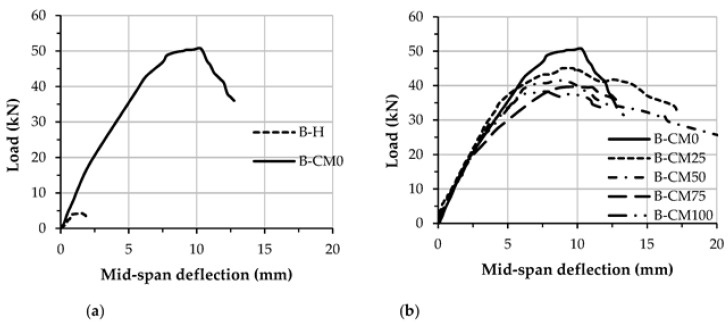
Load vs. mid-span deflection relationship of concrete-filled C-section specimens; (**a**) Hollow and filled specimens; (**b**) Varied contains of recycled aggregates.

**Figure 11:**
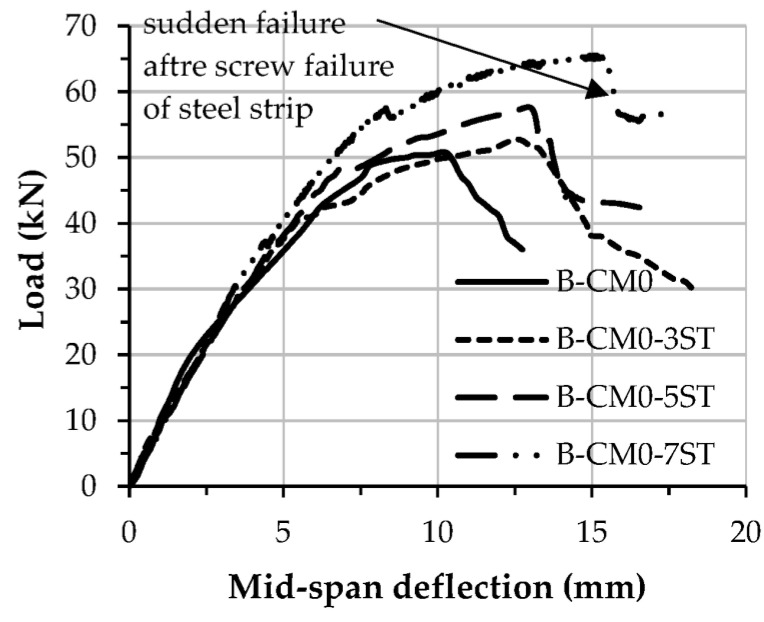
Load vs. mid-span deflection relationship of stiffened specimens with steel strips.

**Figure 12:**
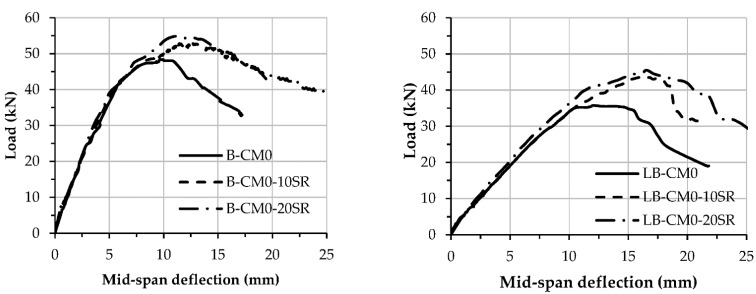
Load vs. mid-span deflection relationship of stiffened specimens with screws.

The flexural load vs. mid-span deflection relationships of the tested concrete-filled C-sections are shown in Figure 10, Figure 11 and Figure 12. Generally, the load–deflection curves start with linear behaviors up to the load yielding limit, which is approximately at the deflection limit of about *L_e_*/300. Then, the load–deflection curves show elastic–plastic behavior up to 80–90% of the specimens’ loading capacities, where steel buckling starts to occur and the width of concrete cracking becomes wider, where after that the load–deflection curves show fully plastic behavior and reduces rapidly till the end of test.

The load–deflection curve of concrete filled specimen B-CM0 showed much better stiffness and ductility behavior than that of the hollow specimen B-H, as shown in Figure 10a. The most interesting outcome for the newly suggested composite members is that the C-sections filled with different percentages of recycled aggregates showed similar load–deflection behavior to that filled with normal concrete (0% recycled aggregates), as shown in Figure 10b. However, the load–deflection curves of the C-specimens filled with recycled aggregates achieved lower flexural stiffness and strength than that filled with normal concrete, this performance is considered reasonable since the lightweight-recycled concrete materials achieved lower compressive strength compared with the normal concrete material, as shown earlier in Table 2.

The load–deflection curves of the specimens stiffened with multi steel strips are stiffer than the control specimen (B-CM0), as shown in Figure 11. Additionally, the load–deflection curve of the specimen stiffened with more steel strips (B-CM0-7ST) behaved stiffer and achieved a higher loading capacity compared to the specimens stiffened with a lesser number of steel strips (i.e., B-CM0-3ST and B-CM0-5ST). The load–deflection curves of the stiffened specimens with steel strips showed a sudden drop in stiffness once they reached their ultimate capacity, because of sudden failures in their steel strip connections and subsequent cracks in the core concrete in the high shear zone. Moreover, the load–deflection curves of the specimens stiffened with multi-screw connectors were compared, as shown in Figure 12, and showed slightly higher load-deflection curves specifically at the elastic–plastic stage than that of the unstiffened specimens (B-CM0 and LB-CM0), this improvement increased continually with the increasing number of connectors.

**Figure 13:**
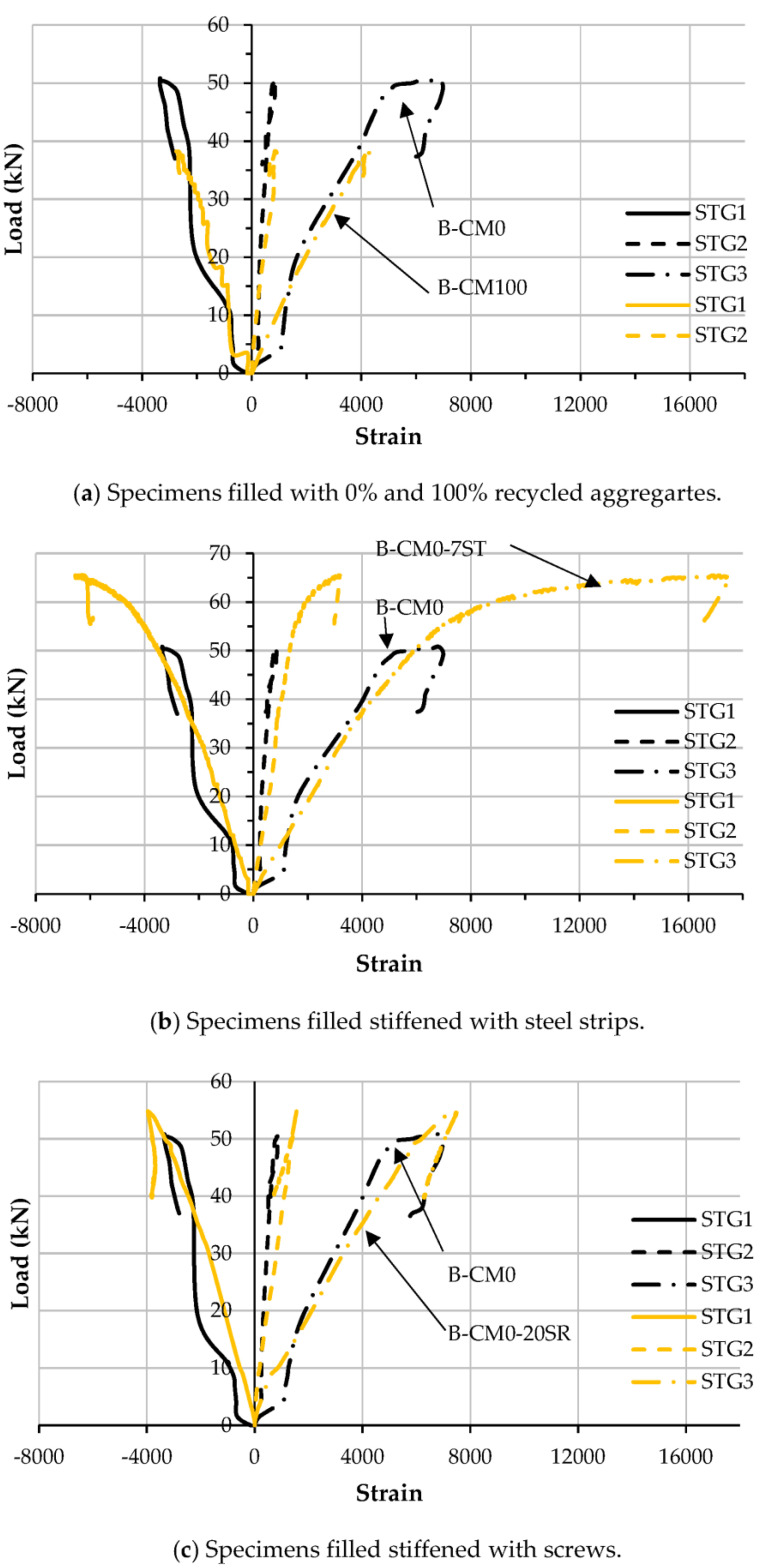
Load vs. tensile strain relationships of tested specimens.

The relationships between the load and longitudinal strain of the steel C-sections at the mid-span of the tested specimens are presented in Figure 13. In general, replacing the raw aggregates in the concrete material did not change the load–strain relationship, even when a high percentage of replacement (i.e., 100%) recycled aggregate was used, as compared with the specimens B-CM0 and B-CM100, as shown in Figure 13a. This performance had been previously recorded for a double C-section that had been prepared as a closed tube section filled with recycled concrete mixtures [12]. Additionally, stiffening the C-section with multi steel strips provided along the open side and/or providing multi-screw connectors along the top-half of the C-section’s cross section led to improved load–strain relationships, since they achieved higher loading capacity than that of the unstiffened specimen (B-CM0), as shown in Figure 13b,c, respectively.

### 3.3. Loading Capacity

This section presents and discusses the achieved loading capacities (ultimate flexural strength capacity) of the tested C-section specimens that were filled with varied contains of lightweight-recycled concrete materials aggregates, including those stiffened with steel strips and screw connectors. The ultimate load (*P_u_*) and ultimate moment (*M_u_*) for the tested specimens are presented and compared in Table 1. Generally, the results show that increasing the percentages of the replacement recycled aggregates achieved lower *P_u_* values than that of the control specimen filled with normal concrete. The 50% lightweight, recycled concrete aggregate mixture (B-CM50) achieved a *P_u_* value equal to 41.5 kN which was about −18.3% less than that of the B-CM0 specimen (50.8 kN), and then the loading capacity was further reduced, up to 38.3 kN (−24.7%), by using a higher replacement percentage of lightweight, recycled concrete aggregate mixture (100% replacement, B-CM100 specimen); however, the loading capacity was still much higher than the *P_u_* value of the hollow specimen B-H (i.e., 4.3 kN). This performance was considered to be reasonable. Although the compressive strength of the concrete mixture was gradually reduced as the percentage of recycled aggregate increased, the recycled aggregate, when used as infill material in single C-sections, still had a significant influence, which was similar to that of double C-section [12].

Furthermore, both of the stiffening scenarios (providing steel strips or screw connectors) adopted in this experimental investigation improved the loading capacity of the single steel C-sections filled with concrete material. As highlighted earlier, tightening the top and bottom C-section’s flanges with multi steel strips achieved better concrete confinement behavior. For example, using three steel strips to stiffen the concrete-filled C-section achieved a *P_u_* value of 52.8 kN (+3.8% higher than the value of the unstiffened specimen B-CM0), and this was further improved to 65.5 kN (+28.9 %) when seven steel strips were used, as shown in Table 1. The same performance was recorded when connectors were added along the top-half of the cross-sections of the concrete-filled C-section specimens, where the loading capacity improved gradually with reduced spacing between these connectors (i.e., using more screw connectors). Lastly, the improvement in loading capacity of the longer specimens stiffened with multi-screw connectors was more obvious (higher percentage) than the shorter specimens, see the *P_u_* values of specimens B-CM0-20SR and LB-CM0-20R (as compared in Table 1).

### 3.4. Ductility Index

**Figure 14:**
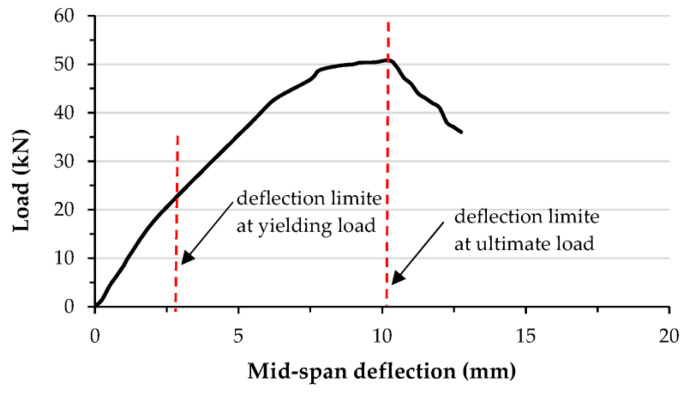
Typical procedure for estimating the ductility index (B-CM0).

**Figure 15:**
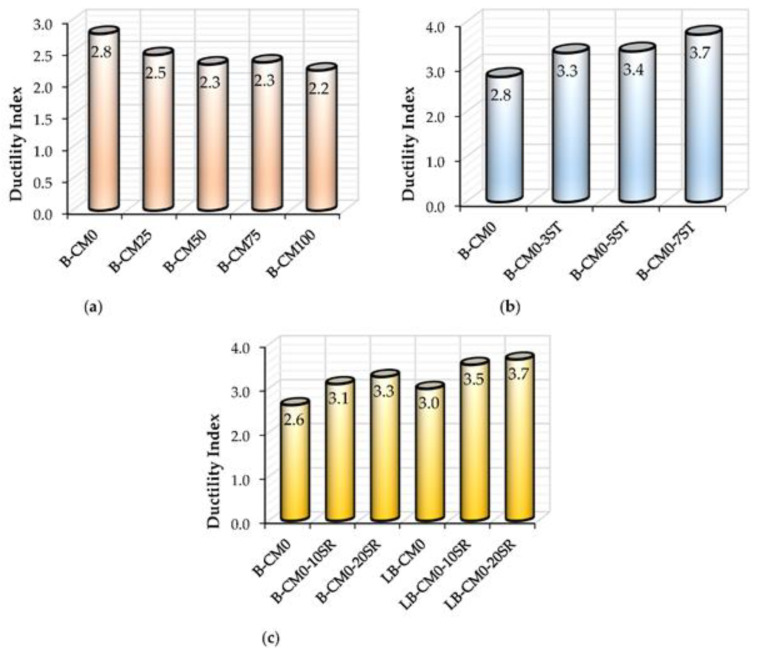
Ductility index (DI) of tested specimens: (**a**) Effects of varied concrete strength; (**b**) effects of steel strips; (**c**) Effects of connectors distances.

This section presents the ductility index (DI) of the tested specimens. The DI performance of the newly suggested composite members is important to be understand in general. For flexural members, the DI is usually estimated using the ratio of the deflection value at ultimate limit (Δ*_u_*) to the deflection value at yielding limit (Δ*_y_*) [50,51], as shown in Figure 14. For the tested C-section specimens filled with a mixture of concrete and different percentages of recycled aggregates, the DI was slightly reduced with increases in the aggregate replacement percentages. For example, the DI for the B-CM0 specimen (i.e., 0% aggregate replacement) achieved a value equal to 2.8, which was reduced about 21.4% (DI = 2.2) when 100% recycled aggregate replacement was used (see Figure 15a), since the ultimate load of specimens with recycled aggregates occurred at an earlier deflection limit than the specimen with normal concrete (0% aggregate replacement), as shown previously by the load–deflection curves. Furthermore, the DI of specimens stiffened with multi steel strips improved gradually with an increase in the number of strips, because the stiffened specimens were significantly improved due to the effects of the steel strips; the stiffened specimen with seven strips (B-CM0-7ST) achieved a DI value of 3.7, which was about 12.1% (DI = 3.3) and 8.8% (DI = 3.4) higher than those stiffened with three and five steel strips (B-CM0-3ST and B-CM0-5ST), respectively, as shown in Figure 15b. Lastly, stiffening the C-sections with multi-screw connectors improved the ductility index, since the related load–deflection curves were improved, as in Figure 15c. Regardless of the span length of the stiffened specimens, since the flexural behavior was improved, then the DI values improved accordingly, thus, the improvement percentage of the DI was increased gradually with an increased number of connectors, for example, as illustrated by the DI values of the D-CM0-10SR and B-CM0-20SR specimens.

## 4. Numerical Method

### 4.1. Describe the FE Model

**Figure 16:**
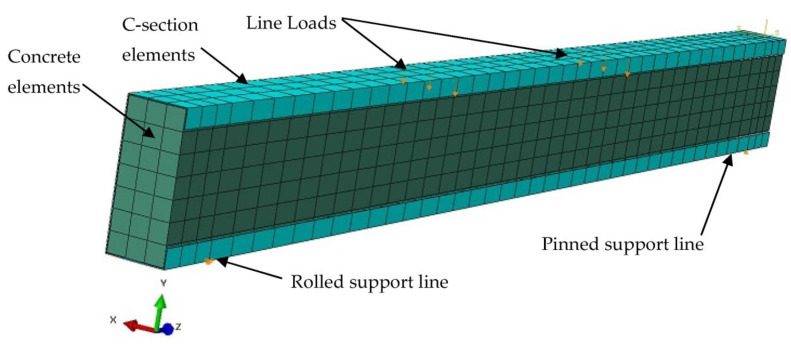
Typical FE model (specimen B-CM0).

The performance of the steel C-sections filled with concrete material is further investigated in this section using a numerical method. The nonlinear finite element software, namely ABAQUS, was used for this purpose. In particular, the tested specimen B-CM0 was simulated, as shown in Figure 16. The loading was applied using line loads located at the top flange of the C-section; the nodes located along these lines were gradually moved downwards during the FE analysis to implement the actual load during testing [11,14,52]. There are two lines located underneath the bottom flange of C-section, which represent the pinned and rolled supports that allow the model to rotate freely around the horizontal x-axis, but they are restricted to the vertical movement (y-axis). The total reaction values accounted from these supports are equal to the total load applied in the FE model [12,14].

**Figure 17:**
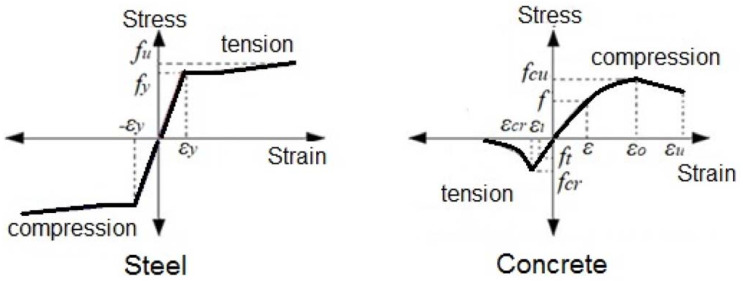
Materials constitutive stress–strain models.

The components of the FE model are the same as the corresponding tested specimen B-CM0, which had infill concrete and steel C-section. These are equal to 40.1 MPa, 26.6 GPa, 489 MPa, 558 MPa, and 201 GPa of the average cubic concrete compressive strength, concrete elastic modulus, steel yield strength, steel ultimate strength, and steel elastic modulus, respectively. The solid element type C3D8R, available in the ABAQUS software’s library, was selected for the concrete elements, while the shell element type S4R was selected for the C-section elements [12]. Based on a preliminary convergence study, a partial interaction between the inner surface of the C-section and outer surface of the core concrete was adopted to select a friction coefficient equal to 0.2. In general, the value of the friction coefficient varies from one case to another, for this type of composite member, it depends on the size, shape, concrete confinement behavior, component properties, and the loading scenarios [6,14,19,53]. Accordingly, the same constitutive models of stress–strain relationships for concrete and steel materials previously adopted in [19,24] were used in the current numerical method, as shown in Figure 17. Concrete material is considered to be a brittle material that is usually crushed and cracked under compression and tensile stresses, respectively. Thus, the concrete damage plasticity option was used to identify the properties of the concrete material [24,52,54]. The elastic–plastic isotropic option was selected to identify the physical properties of the steel material (i.e., elastic modulus, Poisson’s ratio, and yield strength).

### 4.2. Verifying the FE Model

**Figure 18:**
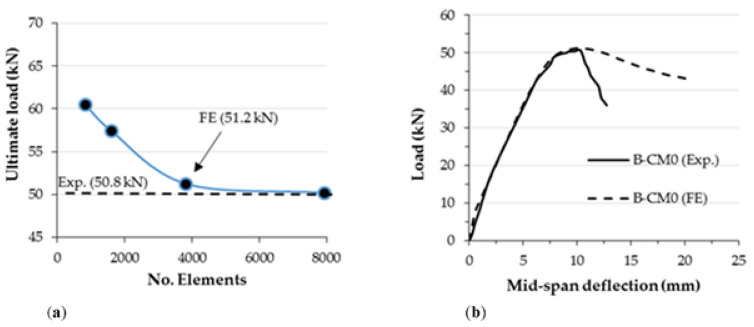
Verifying the results of FE model B-CM0: (**a**) Mesh size convergence study; (**b**) Compare the load-deflection curve.

**Figure 19:**
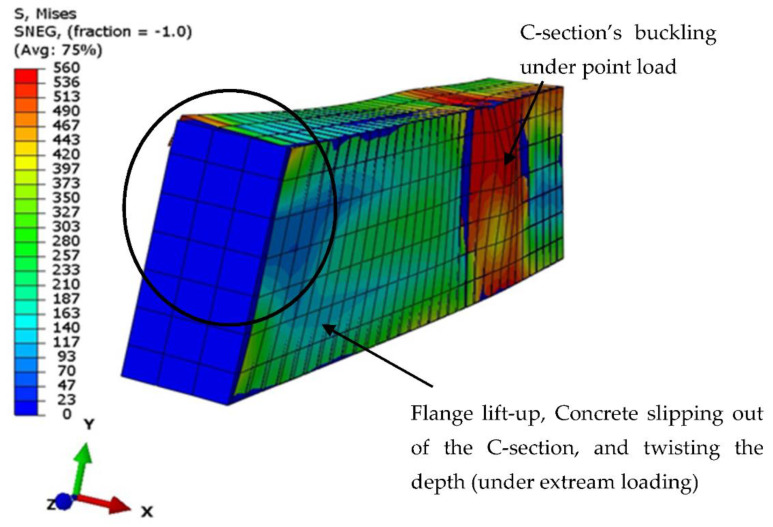
Verifying the failure mode of the FE model B-CM0.

The results of the analyses using the currently developed FE model were verified with the corresponding tested specimen B-CM0, since the same boundary conditions and materials properties were considered. First, a convergence study was performed to select a sufficient mesh size for the elements of the suggested FE model, since increasing the number of elements may not be necessary to achieve accurate results, as shown in Figure 18a. The load vs. deflection curve of the FE model was perfectly matched with the one that experimentally obtained for the corresponding tested specimen B-CM0, as shown in Figure 18b. In addition, the failure mode of the analyzed FE model, including the tube’s outward buckling and twisting failures, and the concrete slipping of the C-section at extreme loading stage, is very similar to the actual failure mode recorded for the tested specimens, both results were reasonably achieved by the currently built and analyzed FE model, as shown in Figure 19.

### 4.3. Parametric Study

Further parameters were investigated after confirming the validity of the proposed FE model of the C-sections filled with concrete, for example, the effects of varied plate thickness and the depth of the C-sections were investigated. Specifically, C-sections with thicknesses of 1.6, 2.0, 2.5, and 3.0 mm were considered (the highest thicknesses available in the local market) which achieved *D*/*t* values of 94, 75, 60, and 50, respectively. In addition, the C-sections with depths equal to 125, 150, 15, and 200 mm achieved *L*/*D* values of 10.8, 9.0, 7.7, and 6.7, respectively. Meanwhile, the rest of the physical properties of the infill concrete and steel C-section remained without change as described earlier in Section 4.1, and all FE models in this section are 1.5 m in total length.

**Figure 20:**
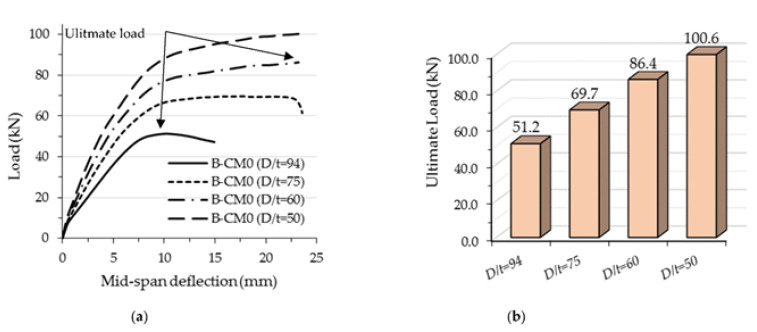
Comparison of the results of FE models with varied depth-to-thickness ratios (*D*/*t*): (**a**) Load-deflection relationship; (**b**) Ultimate load.

**Figure 21:**
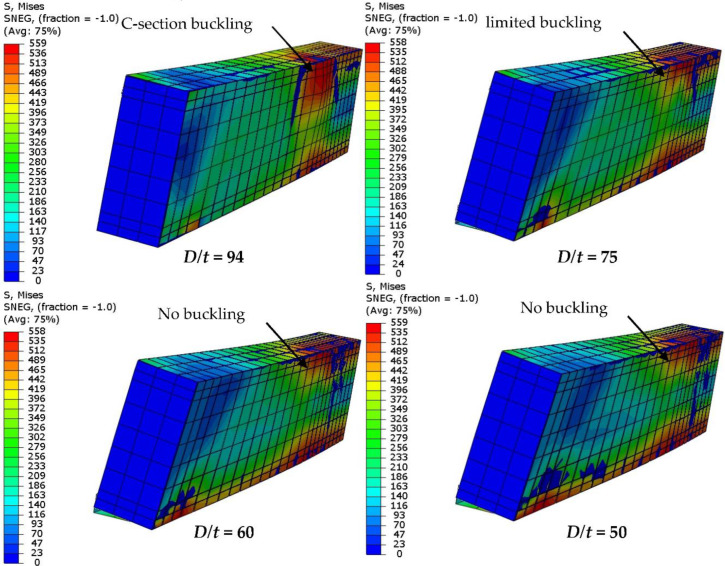
Effects of the *D*/*t* ratio on the steel C-section’s buckling behavior.

The flexural performance of the concrete-filled C-section models changed due to the effects of varied steel thicknesses; the flexural stiffness and strength were both significantly improved with an increase in C-section thickness, as shown in Figure 20. The ultimate load capacity of the analyzed model with a higher *D*/*t* ratio occurred at an earlier deflection limit than those with lower ratios (see Figure 20a). For example, the ultimate load value of the FE model with 1.6 mm C-section thickness (*D*/*t* = 94) achieved 51.2 kN, which occurred at a deflection limit equal to 10 mm, while the same model achieved a higher load capacity of 86.4 kN (+68.7% improvement) when the thickness was increased to 2.5 mm (*D*/*t* = 60), as shown in Figure 20b. This behavior was considered to be reasonable since the outward buckling failure of C-section was gradually restricted with an increase in steel thickness (less *D*/*t* ratio), see Figure 21.

**Figure 22:**
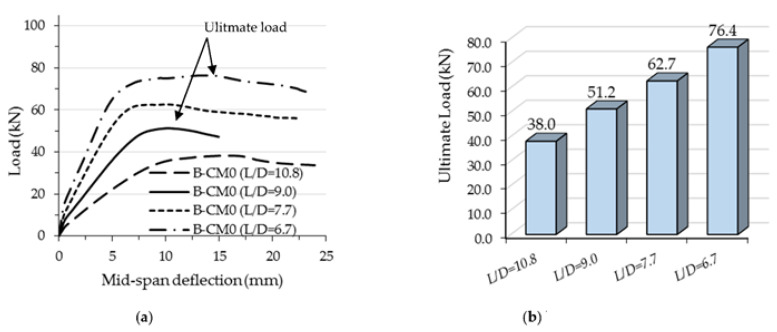
Comparison of the results of FE models with varied length-to-depth ratios (*L*/*D*): (**a**) Load-deflection relationship; (**b**) Ultimate load.

**Figure 23:**
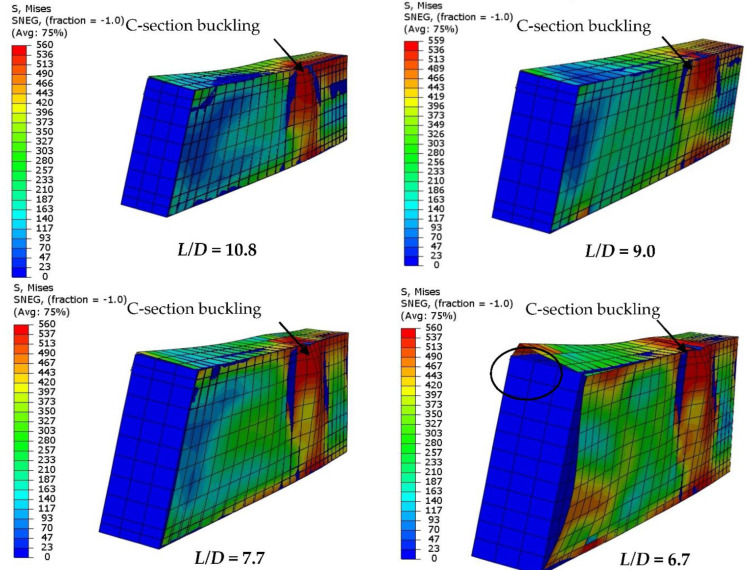
Effects of the *L*/*D* ratio on the steel C-section’s buckling behavior.

Furthermore, as shown in Figure 22, reducing the *L*/*D* ratio of the concrete-filled single C-section model led to increases in flexural stiffness and strength accordingly, since reducing the *L*/*D* means reducing the slenderness ratio of the beam’s span and becoming closer to the non-compact and/or compact span’s limit [12]. For example, the 51.2 kN loading capacity of the control model with *D*/*t* equal to 9.0 (*D* = 150 mm) was significantly improved by about 22.4% (62.7 kN) when the *D*/*t* ratio was reduced to 7.7 (*D* =175 mm). In general, the loading capacity of models with higher *L*/*D* ratio occurred at lower deflection limits than the limit of *L*/50 (see Figure 22a). Figure 23 shows the outward buckling failures of the FE models with varied *L*/*D* ratios, which mainly occurred since there were only slender thickness (1.6 mm) models in this group.

## 5. Conclusions

In this study, the following conclusions have been established based on the results of the experimental and numerical investigation of concrete-filled C-sections under pure bending load:

➢ The flexural loading capacity of the new composite member (single cold-formed steel C-section filled with concrete materials) is significantly increased, i.e., more than 8 times, even by using 100% lightweight-recycled aggregates in the concrete mixture, which are a combination of four different waste materials (RCA, EPS, CRA, and FGA). This is due to the influence of infill concrete material that delayed the buckling/twisting failure of the hollow C-section.
➢ Unlike a double C-section fabricated with closed tube section that was tested by others, the newly tested single C-section cannot achieve high concrete confinement impact, due to how the top and bottom flanges in the single C-section still has one side open along its span. Thus, concrete cracking that usually occurs under two-point loads rapidly increases and the concrete leaks outside the tube section of single C-section at the high bending loads. However, this failure performance can be significantly improved by stiffening the concrete-filled single C-section with steel strips provided along the open side and/or by providing multi-screw connectors distributed equally along the span on the top-half of beam’s cross-section.
➢ It is worthwhile to note that using multi steel strips or screw connectors for stiffening the single C-sections filled with concrete incredibly increased the flexural loading capacity. For example, using seven steel strips to stiffen the open side of C-section or providing 20 multi-screw connectors on the top-half of a C-section’s cross-section increased the loading capacity about 28.9% and 13.4%, respectively as compared with the corresponding unstiffened specimens.
➢ Additionally, the ductility index of the suggested single cold-formed steel C-section beam filled with concrete materials was clearly improved once any of these stiffening scenarios (providing steel strips or screw connectors) was adopted, since bonding behavior between the core concrete and steel C-section parts was significantly enhanced.
➢ Lastly, the numerical model of the specimens of a single C-section filled with concrete material perfectly verified the actual flexural capacity and failure mode of the corresponding tested specimen. The results of the FE analysis confirmed that slightly reducing the *D*/*t* and/or *L*/*D* ratios significantly improved the flexural capacity and behavior of C-sections filled with concrete material. For example, using single steel C-section filled with concrete with *D*/*t* equal to 75 (2.0 mm tube thickness) instead of 94 (1.6 mm tube thickness) led to an increase in the flexural loading capacity of about 36.1%.

The conclusions of this investigation are limited to the size and material properties that were adopted in the current experimental and numerical research. Further investigations are required to adopt newly suggested composite members in future modern projects, including stiffening, strengthening, and connection concepts.

## Figures and Tables

**Table 1:** Designations and results of the tested specimens.

Item	Specimens Designation	Specimens Specification	*L_e_* (mm)	Strip/Screw Spacing (mm)	*Le*/*D*	*P_u_* (kN)	*M_u_* (kN.m)	Strength Deviation (%)
1	B-H	Hollow	1350	-	9	4.3	1.0	-
2	B-CM0	Filled with concrete—0% recycled aggregates	1350	-	9	50.8	11.4	-
3	B-CM25	Filled with concrete—25% recycled aggregates	1350	-	9	45.0	10.1	−11.4
4	B-CM50	Filled with concrete—50% recycled aggregates	1350	-	9	41.5	9.3	−18.3
5	B-CM75	Filled with concrete—75% recycled aggregates	1350	-	9	39.5	8.9	−22.2
6	B-CM100	Filled with concrete—100% recycled aggregates	1350	-	9	38.3	8.6	−24.7
7	B-CM0-3ST	Filled with concrete—stiffened by 3 steel strips	1350	337.5	9	52.8	11.9	+3.8
8	B-CM0-5ST	Filled with concrete—stiffened by 5 steel strips	1350	225.0	9	57.5	12.9	+13.2
9	B-CM0-7ST	Filled with concrete—stiffened by 7 steel strips	1350	168.75	9	65.5	14.7	+28.9
10	B-CM0	Filled with concrete—without stiffeners	1350	-	9	48.3	10.9	-
11	B-CM0-10SR	Filled with concrete—stiffened by 10 screw connectors	1350	135	9	52.8	11.9	+9.1
12	B-CM0-20SR	Filled with concrete—stiffened by 20 screw connectors	1350	67.5	9	54.8	12.3	+13.4
13	LB-CM0	Filled with concrete (long span)—without stiffeners	1850	-	12.3	35.8	8.0	-
14	LB-CM0-10SR	Filled with concrete (long span)—stiffened by 10 screw connectors	1850	135	12.3	43.5	9.8	+21.7
15	LB-CM0-20SR	Filled with concrete (long span)—stiffened by 20 screw connectors	1850	67.5	12.3	45.5	10.0	+27.3

**Table 2:** Proportion of the concrete mixtures (kg/m^3^).

Mixture Designation	Cement (kg)	Fine Agg. (kg)	Coarse Agg. (kg)	Silica Fume (kg)	Water (kg)	Superplasticizer RF 611 (L)			Density (kg/m^3^)	*f_cu_* (MPa)	*E_c_* (GPa)
CM0	395	700	1115	0	190	0			2323	40.1	26.6
CM25	356	630	948	40	190	1.42			2185	38.8	26.2
CM50	356	560	781	40	190	1.42			2166	25.0	21.1
CM75	356	560	502	40	190	1.42			2036	23.0	20.2
CM100	356	525	279	40	190	1.42			2030	22.7	20.1
Mixture Designation	EPS (%)		EPS (kg)	RCA (%)	RCA (kg)	CRA (%)	CRA (kg)	FGA (%)	FGA (kg)	Total Agg. Replacement (%)	
CM0	0		0	0	0	0	0	0	0	0	
CM25	15		1.1	0	0	10	26.8	0	0	25	
CM50	15		1.1	15	143	10	26.8	10	54	50	
CM75	25		1.8	30	285	10	26.8	10	54	75	
CM100	25		1.8	50	476	12.5	33.4	12.5	68	100	

## Data Availability

Data are presented in the article.

## Abbreviation

Depth of C-section (*D*), deflection value at ultimate limit (Δ*_u_*) to the deflection value at yield limit (Δ*_y_*), modulus of elasticity for concrete (*E_c_*), modulus of elasticity for steel (*E_s_*), concrete cube compressive strength at 28 days (*f_cu_*), ultimate tensile strength of steel (*f_u_*), yield tensile strength of steel (*f_y_*), flexural stiffness of composite section (*K*), ultimate bending moment capacity (flexural strength capacity; *M_u_*), effective length (*L_e_*), steel C-section thickness (*t*), width of C-section (*W*), depth-to-thickness ratio (*D*/*t*), length-to-depth ratio (*L*/*D*).
